# “When people see me, they know me; they trust what I say”: characterizing the role of trusted sources for smoke risk communication in the Okanogan River Airshed Emphasis Area

**DOI:** 10.1186/s12889-022-14816-z

**Published:** 2022-12-20

**Authors:** Leah M Wood, Savannah M D’Evelyn, Nicole A Errett, Ann Bostrom, Cody Desautel, Ernesto Alvarado, Kris Ray, June T Spector

**Affiliations:** 1 Department of Global Health, University of Washington, Seattle, USA; 2 Daniel J. Evans School of Public Policy and Governance, University of Washington, Seattle, USA; 3 Department of Environmental and Occupational Health Sciences, University of Washington, Seattle, USA; 4 Confederated Tribes of the Colville Reservation Natural Resources Department, Nespelem, USA; 5 School of Environmental and Forestry Sciences, University of Washington, Seattle, USA

**Keywords:** Risk communication, Public health, Wildland fire, Smoke, Trusted sources, Trust, Environmental hazard, Thematic analysis, Rural health, Tribal health

## Abstract

**Introduction:**

As wildfire smoke events increase in intensity and frequency in the Pacific Northwest, there is a growing need for effective communication on the health risks of smoke exposure. Delivery through a trusted source or intermediary has been shown to improve reception of risk communication messages. This is especially salient in rural and tribal communities who may be hesitant to trust information from state and federal agency sources. This study aims to identify and characterize trusted sources for smoke risk information in the Okanogan River Airshed Emphasis Area (ORAEA), a rural region of North Central Washington state that is heavily impacted by smoke from wildfires and prescribed fire.

**Methods:**

The research team conducted a qualitative study using data collected through key informant interviews and focus groups to assess the role of various sources and intermediaries in disseminating smoke risk information. We used a consensual coding approach in NVivo Qualitative Analysis Software to sort data into preliminary categories, which were grouped into themes using a thematic analysis approach. We used member checking and iterative feedback processes with local project partners throughout the project to ensure credibility of results.

**Results:**

Through the analysis, we identified three themes characterizing trusted sources for smoke risk communication in the ORAEA. These themes were: (1) local and tribal sources of information are perceived as more trustworthy than state and federal government sources, (2) trustworthiness is determined by an evaluation of multiple factors, in particular, perceived credibility, quality of information, and relationship with the source, and (3) conservative political ideology and perceived parallels with COVID-19 communication influence perception of trust. Within each theme, we identified several sub-themes, which contributed additional nuance to our analysis.

**Conclusion:**

This study provides insights into which sources of information are trusted by rural and tribal community members in the ORAEA and why. Results from our study emphasize the importance of relationships and collaboration with local and tribal partners in smoke risk communication. In this paper, we discuss implications for state and federal agency practitioners and present recommendations for how to work with local and tribal partners on smoke risk communication.

## Introduction

Wildfire plays an essential role in maintaining the ecological health of landscapes in the Pacific Northwest and other western coastal states, with many ecosystems having adapted to require periodic fire for regeneration [1, 2]. In the past few decades, however, wildfires have grown in intensity and frequency. This change has been exacerbated by the impacts of over a century of federal and state policy prioritizing fire suppression and by the impacts of climate change [3–7]. Smoke from large wildfires represents a growing public health threat, contributing to an estimated 6,300 annual deaths in the United States [8].

In Washington state (WA), rural and tribal communities in the Okanogan River Airshed Emphasis Area (ORAEA), which includes tribal and non-tribal land within and surrounding the towns of Omak and Okanogan, as well as the western half of the Colville Reservation, are increasingly impacted by smoke from wildfires. In addition, this area has been designated as ‘high priority’ for increased prescribed fire by the WA Department of Natural Resources, which will increase smoke in the Spring and Fall seasons for the region [9, 10]. Smoke is not a new phenomenon in this region, as Indigenous peoples, including the Confederated Tribes of the Colville Reservation (CTCR), have continuously used fire to manage local wildfire-prone landscapes where they have lived for millennia [11–14]. Growing development in the Wildland-Urban Interface and the increasing frequency and intensity of large wildfires in the Pacific Northwest, however, mean that more people are being exposed to potentially hazardous levels of smoke than before [8]. Since 2000, there have been 24 nationally declared fire emergencies in Okanogan County, fifteen of which have occurred in the past ten years [15]. In 2021, the ORAEA and neighboring Methow Valley were identified as among the most smoke-impacted areas of Washington [16]. Additionally, systemic inequity exacerbates the burden of wildfire smoke on communities, including those in the ORAEA, impeding the ability of community members to adapt to prolonged wildfire smoke [17].

Smoke from wildfire and prescribed fire contains a number of compounds harmful to human health, including fine and coarse particulate matter (PM), carbon monoxide (CO), methane, nitrous oxide (N_2_O), nitrogen oxides (NOx), and volatile organic compounds (VOC), among others [1]. Short-term exposure to high levels of wildfire smoke is associated with respiratory and cardiovascular impacts, with growing evidence pointing towards an association with all-cause mortality [1, 8, 18–21]. The risk of health impacts is highest among older adults, children, pregnant people, and people with chronic health conditions [18]. While wildfire smoke and prescribed fire smoke exhibit different risk profiles in terms of smoke composition, concentration, and the ability to mitigate exposure and impact through planning, both forms of smoke are potentially harmful to individual and community health [21]. The long-term impacts of wildfire smoke exposure are not yet well characterized; however, studies demonstrating the impacts of PM from other sources show associations with increased respiratory and cardiovascular morbidity and mortality, among other impacts [22]. There is limited research on the mental health impacts of smoke exposure. A small number of studies, however, including a study set in the Methow Valley, WA, have indicated that smoke exposure may be associated with anxiety, depression, social isolation, and other mental health impacts [23, 24].

The scale of wildfires is expected to increase under current climate change projections [5], and federal, state, and tribal governments are expanding efforts to scale up the implementation of prescribed fire to mitigate the growing impacts of these wildfires [9, 21]. At the same time, there is an increasing need to understand how to best communicate risk in advance of, during, and after smoke events, and to encourage people to adopt interventions to reduce personal risk. Individual-level interventions, such as using high-efficiency particulate air (HEPA) filters or wearing respirators, can reduce risk of respiratory and cardiovascular health impacts [19], but it remains unclear if and how wildfire smoke risk communications support their adoption. In addition, failure to adopt individual-level interventions may not be the result of misunderstanding or not receiving risk communication messages, but may, rather, be attributable to contextual factors such as social vulnerability or access to resources needed to take action [17, 25, 26]. Evidence suggests the use of health promotion frameworks such as the Health Belief Model (HBM) and Theory of Planned Behavior (TPB), which identify motivating factors and perceived benefits and barriers to behavior change, may be effective in identifying and addressing motivating factors for adoption of these interventions [27–29]. The HBM asserts that behavior change is dependent on perceptions of risk, benefit, barriers, self-efficacy, and a cue to action, and has been used extensively in health promotion [28, 30]. Similarly, the TPB emphasizes attitudes about the behavior and response, perceived norms around the behavior, and perceived power and control over the behavior [29]. More research is needed, however, on these applications specific to wildfire and prescribed fire smoke [31, 32]. The few evaluations of wildfire smoke risk communication that exist are focused on emergency messaging rather than long-term preparation and response, and rarely include consideration of prescribed fire smoke [33–35].

The Social Amplification of Risk Framework (SARF) characterizes the social context and processes through which information is received, understood, and transmitted throughout a community or population, thus contextualizing individual risk perceptions and perceived social norms, which are outlined in the HBM and TPB [36–38]. Trust in the source of information influences if and how a message catches the audience’s attention, is believed, and influences action (including continuation of communication) as a result of this information [39–42]. Information delivered through a trusted source or intermediary is more likely to result in behavior change or changed perception, supporting the idea that effective communication is determined not just by what is being communicated but by whom [33, 43].

Existing literature on the role of trust in environmental hazard risk perception suggests the delineation of two forms of trust: social trust, which is based on the relationship between the trusting person and the other, and confidence, which is based on past experiences and behaviors of the other [42, 44]. These different types of trust have varying implications for risk perception, depending on the situation. Additionally, a person’s values, knowledge, and attitudes can influence from which sources they seek information and form trust [37, 39, 45]. This search process has been influenced by increased use of social media, which allows members of groups and online communities to quickly share information and discuss with trusted peers, though information is not always factual or accurate, further amplifying information throughout social networks [39].

Conventional sources of smoke risk information, such as federal and state government organizations, scientists, and mainstream media, may not be perceived as trustworthy by some, thus undermining the salience of the information and limiting the reach of their messaging [39, 46, 47]. This is especially true for rural and tribal communities, who, for a number of reasons, including governments’ failure to recognize tribal sovereignty under treaty rights and systemic disinvestment and marginalization, may be hesitant to extend trust to certain government agencies as sources of risk information [43, 46, 47]. The lack of trust in government agencies is not surprising; a Pew Research Center survey found that only two in ten Americans report trusting the federal government to make the right decisions “just about always” (2%) or “most of the time” (19%) [48] and, in another survey, a majority of respondents view distrust of the federal government and each other as interfering in solving societal problems [49]. Important to note, however, is that public perception of declining trust has often been greater than actual reported declines in trust [49].

While there are significant gaps in the literature around trusted sources and intermediaries for the dissemination of wildfire smoke risk information; recent literature on risk communication related to COVID-19 builds upon an existing body of literature suggesting the potential of leveraging partnerships with community-based organizations and trusted individuals and groups to further amplify risk communication messages throughout a community [39, 40]. Success through community air quality monitoring networks [50, 51] and other community science projects [32] further supports an emphasis on community-based co-production and this leveraging of local social networks for dissemination of environmental hazard risk communication [47, 52–54].

Through this study, we aim to address the gaps in the wildfire and prescribed fire smoke risk communication literature by: *(1) identifying and characterizing trusted sources for smoke risk communication in the ORAEA* and *(2) identifying opportunities to effectively engage trusted individuals, groups, and networks in the ORAEA as potential sources or intermediaries for smoke risk communication.* Results from this study can inform smoke risk communication practice in the ORAEA and other smoke-impacted rural and tribal communities in the Pacific Northwest region.

## Methods

### Study design

The research team conducted a qualitative study using key informant interviews (KIIs) and focus group discussions (FGDs) to assess the current and potential role of various sources and intermediaries for disseminating smoke risk information throughout communities.

### Study setting

This study takes place in the ORAEA, which is located along the Okanogan River Valley in North Central WA. The region is home to roughly 19,500 people, most of whom are clustered in or around the towns of Omak and Okanogan. 56% of people living in the ORAEA identify as white, 25% as American Indian or Alaska Native, 14% as Latino, 4% as two or more races, and less than 1% as Asian or Black [55]. The median household income for this area is $47,299, compared to the WA average of $73,755. This area includes the western region of the Colville Reservation, where most people are enrolled members of the CTCR, descending from one or more of twelve bands whose traditional territories extend across much of North Central Washington, crossing into British Columbia and parts of Oregon and Idaho [55, 56].

We chose the ORAEA as the location for the parent study because of the high burden of smoke on communities in the area and because of the long history of tribal burning and prescribed fire for land management purposes. To this day, the CTCR continue to use fire as a key part of their strategy for land stewardship and are active leaders in the intertribal advocacy movement for tribal burning rights [11, 57]. Communities in the ORAEA are routinely exposed to high levels of smoke from frequent wildfires and prescribed fire and rank among the top 10–20% of census tracts in Washington State for exposure to fine PM [55]. An analysis of air quality data from the Omak EPA Air Monitoring Station showed that 41% of days in 2021 had an average daily air quality ranking of ‘moderate’ or worse [58, 59]. In addition, most census tracts in the ORAEA are considered “highly impacted,” or ranked in the top 10–20% of census tracts in Washington State, by social vulnerability to hazards, exacerbated by high rates of poverty and unemployment [55].

We collected data for this study through a series of 17 KIIs and six FGDs. KIIs were held virtually or over the phone between June and November 2021 and FGDs were held in person in October and November 2021 in Nespelem and Omak, WA.

### Sampling strategy

We recruited participants for KIIs using a combination of purposive and snowball sampling and included people who (1) were year-round residents of the ORAEA, (2) were ages 18 years or older, and (3) self-identified as community leaders and/or public service organization staff or volunteers with experience working in local government, with elders, with schools and/or youth organizations, in public health roles, in emergency management, in forestry or agriculture, and/or communicating about air quality.

In collaboration with our community partners, we developed a list of stakeholder categories from which to recruit interviewees and identify targets for recruitment (Table 1). Community partners identified potential contacts from within the community who could speak to the research questions and sent introductory emails with information about study aims and participation. We made an initial 36 contacts, resulting in 17 KIIs. Our community partners identified a greater number of non-tribal contacts (*n* = 24) than tribal (*n* = 12). A similar response rate from non-tribal contacts (46%) and tribal contacts (50%) resulted in a higher number of non-tribal interviews. KIIs were held until thematic saturation of meta themes was reached, meaning that theme redundancy was observed across KIIs and it was perceived unlikely that additional data collection would lead to significant new insights [60]. For both KIIs and FGDs, participants were offered a $50 Tango or VISA gift card as compensation.

**Table 1:** Key Informant Interview Target and Actual Sample Frame

Stakeholder Category	Target	Tribal Interviews	Non-Tribal Interviews	Total	
People with significant firsthand work and/or volunteer experience in **local government leadership**	*2–3*	1	2	3	
People with significant firsthand work and/or volunteer experience with **elders**	*2–3*	1	2	3	
People with significant firsthand work and/or volunteer experience at or with **schools and/or youth organizations**	*2–3*	1	1	2	
People with significant firsthand work and/or volunteer experience in **public health or healthcare**	*1–2*	1	2	3	
People with significant firsthand work and/or volunteer experience communicating about **air quality**	*1–2*	1	2	3	
People with significant firsthand work and/or volunteer experience in **emergency management**	*1–2*	1	1	2	
People with significant firsthand work and/or volunteer experience in **agriculture and/or forestry**	*1–2*	0	1	1	
**TOTAL**	*16*	6	11	17	

We recruited FGD participants using purposive sampling and included people who were: (1) year-round residents of the Okanogan River Valley and/or the Colville Reservation and (2) ages 18 years or older, or 16–18 years old with parental consent. FGDs were structured into three age groups: youth and young adults (ages 16–25), elders (65+), and mixed-generation community members. FGDs were structured in this way to examine how information sources and absorption may vary by age and to allow dynamics between participants to help shape the data [61, 62]. We recruited FGD participants using a combination of recruitment by community partners, social media posts on community Facebook pages, and flyers around Omak and Okanogan, WA.

### Data Collection and Study Instruments

To address study aims we developed a guide for the KIIs, which we then refined by integrating feedback from community partners, to ensure local and cultural relevance. Using this guide, we held a total of 17 one-hour KIIs with community leaders and people in public service roles. KII participants were asked to describe their perceptions of how information about smoke is communicated. In the KIIs, they provided formative insight into community and cultural values and perspectives on smoke from wildfires and prescribed fires, as well as smoke communications.

Information learned through KIIs subsequently informed FGD protocol development, which also integrated community partner feedback. Two ninety-minute FGDs targeting non-tribal community members in two different age groups, and four targeting tribal community members and CTCR tribal employees in three different age groups, asked participants to describe if and how they receive and share information on the health risks of smoke. FGD participants were also asked to assess existing sources for relevance and credibility of information and identify gaps and opportunities for improved smoke risk communication. KIIs and FGDs were conducted in English by a graduate student and a postdoctoral researcher at the University of Washington, with assistance from CTCR staff and project team members.

### Data analysis

We audio recorded KIIs using Zoom or Google Voice. Recordings were then de-identified and transcribed verbatim using the TranscribeMe professional transcription service. We audio recorded FGDs using Audacity software, which we then de-identified, adjusted for audio quality, and transcribed verbatim ourselves. During both KIIs and FGDs, we took notes which were then synthesized into overarching themes. We used member checking to assess trustworthiness of interpretation by sending KII participants synthesized versions of notes from their interview and allowing interviewees to respond with feedback [63, 64]. Of seventeen interviewees, five returned feedback, for a response rate of 29%. Each set of notes was then organized by research question and research question-level summaries, including edits provided through member-checking, were plotted onto a matrix (i.e., table). This process facilitated the identification of emerging themes and counterpoints across KIIs, which were used to inform inductive code generation.

We used a combined deductive and inductive approach to construct a codebook for the parent project, with concepts from the HBM and TPB driving the deductive phase. Themes identified through the above data familiarization process and thorough review of KII and FGD transcripts were used to identify inductive codes. We used an iterative approach to codebook development, revisions, and clarification to approach high interrater reliability. This approach included co-coding four transcripts, reviewing areas of discord, refining the codebook, and repeating this process until codes were applied consistently between coders [65]. We used the same codebook to code both KII and FGD data, as KIIs and FGDs considered similar topics and themes; however, we used labels to distinguish between excerpts from KIIs and FGDs and to support comparative analysis. Data were coded using NVivo qualitative analysis software and reviewed by a second study team member. We used a second round of coding to further organize the data into more specific subcodes related to trust and trustworthiness.

We applied the framework method to completing a thematic analysis of the data, plotting summarized data for each code on to a matrix, organized by KII and FGD [66–69]. Data for each code were synthesized and grouped by tribal data, non-tribal data, and aggregate data to support comparison between cases. We then used network diagrams to further explore relationships between codes [70]. As preliminary themes were identified, we collated coded excerpts by draft theme, read and re-read excerpts under each theme, and further refined themes, repeating iterations of this process until themes and sub-themes were clearly defined [66]. We then presented our results to CTCR and ORAP project team partners for feedback, which was integrated into development of final themes and subthemes.

#### Research team positionality

As discussed by Berger (2015), reflexivity is a process of continual self-evaluation and reflection of researcher positionality and how it may influence the research process and outcomes [71]. Our team was composed of researchers from the University of Washington and CTCR staff and community members, and as such hold a variety of identities as relevant to this study. All members of the team have experience as researchers or practitioners in the fields related to wildfire management or air quality, and as such, likely brought assumptions based on our previous work to this project. To account for potential biases and blind spots, we integrated member checking to confirm interpretation of interview notes [63]. We also used an iterative feedback process with team members from the CTCR, who have direct lived experience relevant to this study, throughout the study to ensure credibility in data analysis and interpretation.

## Results

KII and FGDs participants described individuals and groups that they perceived as trusted sources of smoke risk information in their community and why these sources were viewed as trustworthy. Several themes emerged from these conversations which are summarized in Table 2 and discussed in the following section. These themes address our first of two study aims, to *identify and characterize trusted sources for smoke risk communication in the ORAEA.* The second aim, to *identify opportunities to effectively engage trusted individuals, groups, and networks in the ORAEA as potential sources or intermediaries for smoke risk communication*, is addressed in the Discussion and Implications for Practice sections later in this report.

**Table 2:** Summary of Themes

Theme	Sub-Theme
Local sources of smoke information are viewed to be most trustworthy.	Tribal Government (T) Local Government Informal Networks Social Media
Trustworthiness is determined by evaluation of multiple factors.	Credibility Quality of Information Relationships
Political ideology has a strong influence on which sources people will trust.	Political Conservatism (NT) Parallels with COVID-19 (NT) Relationships as Bridges

### Theme 1: Local sources of smoke information are viewed to be most trustworthy

Across KIIs and FGDs, we found that most participants shared that they viewed local sources of information as the primary and most trusted sources of smoke risk information for members of their communities. Local sources included various agencies within local and tribal government, as well as non-governmental and community-based organizations, groups, and individuals within communities in the ORAEA. The majority of participants reported that state and federal governments are generally less trusted, resulting in part from perceived distance from local concerns, differences in political ideology, lack of accountability, and negative past experiences. These include experiences of discrimination and perceived mismanagement of resources. This was communicated most strongly in non-tribal KIIs and FGDs, where almost all participants explicitly referenced perceived distrust of state and federal agencies. Tribal participants tended to state a preference for tribal sources of while sharing neutral feelings towards information from state and federal agencies, though several tribal FGD participants shared anecdotes of perceived mistreatment and mismanagement of resources by state and federal agencies.

### Tribal government

Tribal participants stated that information delivered directly through CTCR agencies and departments, or information with the CTCR logo, was viewed as most trustworthy by tribal members. This information was described as locally and culturally relevant, reliable, and tied into the community. Participants shared that CTCR was viewed as trustworthy because of its vested interest in tribal members’ wellbeing. Several participants reported a growing acceptance of information from state and federal agencies with increased partnership and inclusion of tribes in decision-making; however, information from CTCR remained preferred.

Within the CTCR departments and agencies, the majority of participants shared that the Environmental Trust Department (ETD) and Mt. Tolman Fire Center were viewed as authoritative sources of information on wildfires and smoke. Several tribal participants described seeking information from specific staff who they viewed as respected experts on fire and smoke and who were tribal members themselves. One participant described their experience participating on incident management teams, stating:

*“One thing about the community is that they know the person that’s providing the information, and that’s one of the reasons why they trust me with the incident management teams is to have a tribal member working with the incident management teams so that people would be more trusting in what’s being said and what’s going on out there. So when people see me, they know me; they trust what I say.”* Air Quality Interviewee, Tribal

In this excerpt, the participant details their dual roles as tribal community member and staff participant on incident management teams. As both insider to the community and perceived subject matter expert, these two identities were perceived to positively influence the community’s regard of them as a trusted source of smoke risk information. 

### Local government

Participants shared that local government agencies are generally trusted, as they were perceived as having more tangible impacts on the day-to-day experiences of community members and are thought of as less partisan than state or federal government. Several non-tribal participants described perceived mistrust of specific agencies, however, due to inconsistencies in the information the agency shared. Conservation districts and local fire departments, in particular, were reported as highly trusted because of their authority on fire and perceived neutrality as non-regulatory organizations that work with a variety of partners in the community. Emergency management communications were also described as well-received, as was information delivered through local schools and public utility departments. About half of non-tribal participants shared that while public health agencies were generally trusted, political polarization around COVID-19 had complicated their relationships within communities, an observation discussed in more detail below. Local non-governmental and community-based organizations were highlighted by several tribal and non-tribal participants as being effective at targeted outreach to subpopulations at higher risk of health impacts from smoke and at addressing gaps in communication from local government sources.

### Informal networks

Throughout KIIs and FGDs, many participants emphasized the strength of informal networks within both tribal and non-tribal communities in the ORAEA. Participants shared that they viewed community members as likely to trust information received through friends, family, neighbors, and affiliation groups, even if that information was not fully accurate. These networks were described by both tribal and non-tribal participants as especially effective for reaching people living in more remote, rural areas in the ORAEA or who are living off-the-grid and are not connected to internet or phone service. One participant described this, sharing:

*“I think this county is a great place to live in particular because if something does go down, if I was his neighbor ten miles away, he’s hopping in his truck and driving to my house and telling me.”* Mixed Ages Focus Group Participant, Non-Tribal

In this excerpt, the participant describes a sense of community in the ORAEA where people look out for one another, often going out of their way to share information. This sentiment was echoed by several other participants throughout KIIs and FGDs.

### Social media

Many participants, both tribal and non-tribal, described the emergence and widespread use of private citizen-run fire watch Facebook pages that provide crowd-sourced, hyperlocal information on wildfires and smoke in the ORAEA. Participants shared that these groups were generally viewed as trustworthy, as information was perceived to be timely, locally relevant, and disseminated by community members. At the same time, several participants also acknowledged the potential spread of misinformation through these sources.

### Theme 2: Trustworthiness is determined by evaluation of multiple factors

Both tribal and non-tribal participants described credibility, quality of information, and relationships as among the most important characteristics in determining the trustworthiness of a source of information. Within these characteristics, tribal participants identified authority and expertise of the source, accuracy, timeliness of the information, local and tribal relevance, and relationships as important when evaluating the trustworthiness of a source. Non-tribal participants identified perceived political neutrality, transparency, authority, and respect as important.

#### Credibility

Credibility was described as determined primarily by the source’s perceived authority or expertise on the topic of smoke and fires as well as its transparency, political neutrality, and accountability to communities for their actions. Conversely, participants shared that the perception of a source as lacking in any of these traits was negatively associated with credibility. Public health departments and healthcare workers were described by several participants as having authority and expertise on the health impacts of smoke. Fire departments, including Mt. Tolman Fire Center, as well as conservation districts and the CTCR ETD, were highlighted by both tribal and non-tribal participants as being highly credible, due to their authority and relevant expertise on wildfires and smoke, perceived ‘lack of agenda’ and non-regulatory status, and being firmly rooted within communities. One participant described this, stating:

*“Local fire chiefs or even I think there [are] county health officers or even your local physicians, because I think they’re recognized within the community as having—holding a certain position. And if they’re giving information, I think those people tend to take it a little bit more seriously.”* Air Quality Interviewee, Non-Tribal

Here, the participant describes how the perceived authority of local fire chiefs, health officers, and healthcare workers influences community members’ responses to information shared. Important to note, however, is that not all participants shared this view of public health and healthcare workers due to their association with COVID-19 and the perceived political polarization of the COVID-19 response.

### Quality of information

Participants shared that information needs to be perceived as reliable, accurate, timely, and locally relevant to be considered high quality. Information from local and tribal government agencies and departments was described as well-received by many, especially information shared by CTCR’s ETD. Several tribal and non-tribal KII and FGD participants shared that a lack of locally accurate air quality and weather information negatively impacted their perception of the quality of information shared by local agencies, as did slow communication and availability of information during wildfire and smoke events. A few participants shared that getting information from traditional media and news sources was common, but that it was limited in its relevance, as local news options are limited. Many tribal and non-tribal participants described the popularity of Fire Watch Facebook groups, which provide hyperlocal information in close to real time and filled gaps left by more official channels. While most participants that described these groups shared positive perceptions of the quality of information provided, a few participants cautioned that misinformation is common on social media.

### Relationships

Participants described relationships, respect for, and experience with a source as central to forming trust. Participants shared that most people in their communities seemed to trust people that they knew and saw within their community. Almost all participants described tribal and non-tribal communities in the ORAEA as small and tight-knit, and several shared a perception that community members tended to have some degree of distrust of outsiders. Participants shared that community-based organizations and local agencies that work in a coalition or partnership model had a greater reach than groups working in isolation, as greater numbers of partners translated into greater opportunities for community members to develop positive relationships and interactions.

Several tribal and non-tribal participants stated the importance of partnership and inclusion in decision-making when working with outside government agencies, with one tribal participant sharing:

*“I think that the tribal membership has grown to pretty much appreciate the help that we get from the EPA and the Department of Ecology and other local agencies when the situation comes up to where they’re more trusting than they used to be because now, we have a place at the table where we can voice our opinions. And there was a time when that didn’t happen. But now, we’ve been asked to participate in a lot of important events that are going on, and so their relationships have grown quite steadily.”* Air Quality Interviewee, Tribal

Within this excerpt, the participant describes intentional efforts by agencies to build relationships and share decision-making power as central to forming trust, especially when working with outside agencies and within government-to-government relationships such as those between tribes and state and federal agencies.

### Theme 3: Political ideology has a strong influence on which sources people will trust

In non-tribal KIIs and FGDs, participants described their perception that political ideology influenced how community members determine trustworthiness and trust in certain sources. Several participants drew parallels between political polarization around COVID-19 and smoke risk communication, explaining how political ideology has shaped receptiveness to health messaging around taking action to prevent the spread of COVID-19, including vaccines. Lastly, several participants shared how strong relationships and trust in the source or intermediary can help bridge ideological divides when communicating about sensitive topics such as wildfire smoke.

### Political conservatism

In non-tribal KIIs and FGDs, participants described their perception that political ideology influenced how community members determine trustworthiness and trust in certain sources. While participants emphasized a diversity of opinions in the ORAEA, over half of non-tribal interviewees described Okanogan County as a majority conservative area, or “red county,” and reported that non-conservative government leaders or agencies and media entities were perceived to be less trusted. One interviewee described this, sharing that:

*“Federal government, least, and state government, it’s—we’re a very red county and so whoever’s in power in the legislature, if they’re not red, they’re not trusted at all. State agencies, there’s a lack of trust for state agencies as well.”* Leadership Interviewee, Non-Tribal

In this excerpt, the participant describes a perceived lack of trust in state and federal decision-makers and agencies who do not align with conservative political ideologies and causes. This was echoed by several other participants who shared that non-conservative state and federal agencies may be perceived as not being aligned with residents’ experiences or as having a political agenda.

### Parallels with COVID-19

In non-tribal KIIs and FGDs, participants also highlighted the perceived parallels between communication about COVID-19 and communication about the health impacts of smoke. A few participants shared that masking to protect against the health impacts of smoke was associated by many with masking to prevent the spread of COVID-19, which has been politically polarized in recent years and subject to the spread of misinformation. At the same time, other participants noted that wearing masks during smoke events has become more common with the normalization of masking for COVID-19. Similarly, a few participants discussed the complication of community perceptions of public health agencies and healthcare workers, because of their association with COVID-19 and vaccines, and perceived lack of transparency around these topics. Other participants described these agencies as well positioned to act as authoritative sources for health information about the risks of smoke exposure.

### Relationships as Bridges

Many tribal and non-tribal participants shared their perception that community members in the ORAEA were more likely to trust information shared by friends and family, people in the community with whom they had relationships and shared values, over official sources that may conflict with their worldviews. Participants emphasized the importance of strong relationships in communication and described the opportunity to leverage relationships and well-respected community members in bridging ideological differences around topics related to smoke and health. One participant shared an example of this:

*“We had one wildfire that makes me think about that, the Cold Springs Fire, and the fire chief is also a rancher and we assembled at his ranch to talk about the fire. He emphasized the health issues, rather than the COVID issues, because of the stigma about how people are responding to the COVID. But he did make a statement that no matter what, your health is important for being resilient in recovering from these fires.”* Leadership Interviewee, Non-Tribal

Here, the participant describes how the fire chief leveraged his authority and multiple roles to connect with community members around the sensitive issue of wildfire smoke. He utilized his insider knowledge and relationships within the community to frame the issue in a way that they were receptive to.

## Discussion

The findings of this study are consistent with much of the existing literature on environmental hazard risk communication in rural and tribal communities. Our results emphasize the integral role of trusted individuals and networks in amplifying risk communication messages, as well as a preference for information sourced from ‘within’ the community, both of which have been described in other settings and types of risk communication and explored in SARF [33, 39, 43, 47, 72]. Our study also identified factors influencing trust, including perception of a source’s credibility and the quality of its information, as well as one’s relationship with the source, which align with existing frameworks examining the role of social trust and confidence in risk management and communication [39, 42, 44]. Our study is one of only a few studies that have evaluated, using qualitative methodology, smoke risk communication [35, 52, 73], and to the best of our knowledge, is among the first to examine the role of trust in smoke risk communication within the context of rural tribal and non-tribal communities in the Western United States.

The qualitative approach to our study allowed us to characterize in rich detail not only who is most trusted but also why, for community members in the ORAEA. Our study found that relationships were central to forming trust, which aligns with existing theories of social trust [42] and best practices in environmental risk communication [33, 43, 47]. We found that credibility and quality of information, were also identified as important factors for consideration in determining trustworthiness. For example, local firefighters, because of their positioning as experts and authoritative sources of information on wildfires and smoke, as well as their roles as respected members of the community, are viewed by both tribal and non-tribal residents as highly trustworthy. This insight informs public health practitioners and local decision-makers who can leverage these networks in designing risk communication strategies, potentially expanding the reach and uptake of messages shared.

Our study confirmed that community members’ trust in state and federal government agencies may be tempered by perceived distance between decision-makers and place-based community concerns, negative past experiences, and a lack of transparency or accountability to rural and tribal communities [43, 46, 47, 53]. This is additionally modified by political ideology, which influences how community members construct perceptions of credibility and quality of information [74, 75]. Important to note, however, is that our study examined how community members perceived others’ trust in government agencies rather than examining whether community members trusted government agencies themselves. This may, in turn, reflect an overestimation of community distrust of federal and state government agencies as sources of risk communication messages [39, 49].

Our study found that community members were more likely to trust information shared through informal networks of people within their communities or local and tribal agencies and organizations. This aligns with existing research demonstrating that rather than systematically evaluating individual sources of information, people are likely to use heuristics and look for traits such as shared values, familiarity, and similarities in knowledge, attitudes, and experience to determine trustworthiness [37, 39, 45].This may explain, in part, the proliferation of information shared on social media, which allows for quick dissemination of information through social networks and multi-directional discussion with like-minded peers, allowing for decentralized amplification or attenuation of risk communication messaging [37, 76]. While often positioned as at odds with scientific and government sources of information, social media represent an opportunity to redefine and democratize roles in risk communication amplification, though this is complicated by private ownership of social media and the potentially profit-driven proliferation and spread of misinformation [39, 76–78]. This is happening at the same time as government agencies and official sources of risk information are growing their online presence, further blurring the lines [39].

### Implications for practice

While the findings of this study are specific to the context of rural and tribal communities in the ORAEA, their framing can be applied in other rural and tribal communities to support effective smoke risk communication and meaningful community partnerships. This grows increasingly urgent, as wildfires are expected to become more frequent and intense with climate change, and the intensifying smoke impacts to communities represents a growing public health crisis [3, 5, 8]. Smoke-impacted rural and tribal communities, such as those in the ORAEA, are bearing the earliest and most acute impacts [17]; however, it is likely that smoke events will continue to expand to other areas in the future as fires intensify [3, 5, 8].

Our study suggests that state and federal agency practitioners must implement additional strategies to demonstrate accountability to communities, in acknowledgement of perceived harm and disinvestment perpetrated by government agencies in rural and tribal communities. These principles apply outside of risk communication to equitable community engagement, more generally. With growing attention to the legacies of environmental injustices, federal and state agencies are increasingly required to embed community engagement in environmental policymaking, including around prescribed fire and wildfire smoke management. This is central to policies such as President Biden’s Justice40 Initiative and Washington State’s Healthy Environment for All (HEAL) Act, which aim to address inequities in federal and state agency decision-making processes and the distribution of environmental burdens and benefits, and include mandates for robust community engagement [79–81]. Our study highlighted that engagement with rural and tribal communities must center long-term relationship building, in addition to improving perceived credibility and quality of information shared. Additionally, when working with tribal nations, such as the CTCR, this must also include formal government-to-government consultation processes, and inclusion of tribal nations in state and federal decision-making [82, 83].

Practitioners from state and federal agencies may find working with local and tribal community partners beneficial in their efforts to identify trusted individuals and groups who can act as spokespeople or messengers within their communities. Centering local perspectives and expertise in the design of risk communication messaging can assist in tailoring strategies to improve their relevance and reach [84–86]. This can be especially effective in designing risk communication messaging around politically sensitive topics, such as COVID-19 or prescribed fire. State and federal agency practitioners can support capacity building for local and tribal partners through the provision of unrestricted funding and grants to support smoke readiness, employment of community members in smoke risk communication strategy and implementation, and development of resources that can easily be rebranded with local or tribal agency logos and shared out to community members through their channels. Additionally, agencies can embrace digital messaging and generate content that can be easily shared through online community networks on social media by local partners.

### Strengths and limitations

This study used qualitative methods, which allow for deep, context specific inquiry into how communities in the ORAEA experience the phenomenon of smoke risk communication; however, as these insights were gleaned from a singular community, this approach may limit generalizability of our study’s findings to other rural and tribal communities in the Pacific Northwest. At the same time, due to the complexity of how individuals determine trust and trustworthiness, elements of our study are likely to translate to other settings and the framing of our study may prove useful to others engaging in similar inquiry.

Our study used purposive sampling to identify potential participants whose lived experience and perspective on these issues would allow them to uniquely contribute to our study goals. This sampling strategy also allowed us to leverage the expertise and relationships of members of our study team who, as long-time ORAEA residents and employees of the CTCR, contributed a rich and unique perspective to identifying potential participants. Within this sampling strategy, however, there is opportunity for selection bias. Those with less familiarity or interest in smoke or risk communication, or who are less actively engaged in civic activities, whose perspectives may have been systematically different from those of our participants, may have been excluded from our sample. Additionally, our study was conducted entirely in English, limiting the participation of non-English speakers. Lastly, our positionality as researchers may have impacted participants’ comfort and willingness to share about certain topics, which must be acknowledged in communication of study results.

## Conclusion

Rural and tribal communities in the Pacific Northwest are experiencing growing and prolonged exposure to dangerously high levels of smoke exposure from wildfires and prescribed fire. This is anticipated to grow under current climate projections. Smoke risk communication is an essential part of community resilience to wildfire and prescribed fire smoke, helping to spread awareness of the health risks associated with smoke exposure and motivate adoption of individual-level interventions to mitigate those health risks. Evidence suggests that delivery through a trusted source or intermediary can improve reception of risk communication messages, though few studies have evaluated this for smoke risk communication in the context of rural and tribal communities. These communities may, for a number of reasons including historic disinvestment and marginalization, be hesitant to trust information from state and federal agencies.

In this qualitative study, we conducted a thematic analysis on data collected from KIIs and FGDs with tribal and non-tribal community members in the ORAEA. We identified three main themes centering on the importance of local sources of information; the role of perceived credibility, quality of information, and relationships in determining trustworthiness; and the relationship between political ideology and trust. These themes both align with and extend the existing risk communication literature with rich, qualitative descriptions of not only who is viewed as trustworthy, but why. This study has specific implications for practitioners, particularly state and federal agencies approaching rural and tribal communities as outsiders. Through this report, we present specific recommendations for how to collaborate with local and tribal partners to leverage trusted sources and intermediaries within the community for effective smoke risk communication.

## Data Availability

The data that support the findings of this study are available from the University of Washington and the Confederated Tribes of the Colville Reservation but restrictions apply to the availability of these data, which were used under license for the current study, and so are not publicly available. Data are however available from the corresponding author upon reasonable request and with permission of the University of Washington and the Confederated Tribes of the Colville Reservation.

## Abbreviations

CO: Carbon Monoxide
COVID/COVID-19: Coronavirus Disease 2019
CTCR: Confederated Tribes of the Colville Reservation
ETD: Environmental Trust Department
FGD: Focus Group Discussion
HBM: Health Belief Model
HEPA: High-Efficiency Particulate Absorbing
KII: Key Informant Interview
N_2_O: Nitrous Oxide
NOx: Nitrogen Oxide
ORAEA: Okanogan River Airshed Emphasis Area
PM: Particulate Matter
TPB: Theory of Planned Behavior
VOCs: Volatile Organic Compounds
WA: Washington State
